# Myometrial Cystic Formation after Local Methotrexate Application into Cornual Gestational Sac: A Case Report of an Unexpected Complication

**DOI:** 10.1155/2011/619094

**Published:** 2011-07-09

**Authors:** Zehra Sema Ozkan, Banu Kumbak, Ekrem Sapmaz, Mehmet Simsek

**Affiliations:** ^1^Department of Obstetrics and Gynecology, Firat University School of Medicine, Elazig, Turkey; ^2^Fırat Universitesi Tip Fakultesi Kadin Hastaliklari ve Dogum Ana Bilim Dali, 23119 Elazig, Turkey; ^3^Maternity and Women Health Hospital, Adana, Turkey

## Abstract

Cornual pregnancy is a rare type of ectopic pregnancy, and diverse therapeutic options exist for the management. Medical treatment despite high initial beta HCG values is not thought to be safe. We reported a 39-year-old woman with an initial beta HCG value of 22000 mIU/mL and diagnosed of a cornual pregnancy. Patient was managed successfully with the administration of combined systemic and ultrasonographically guided local injection of methotrexate into the gestational sac. During followup with serial beta hcg measurements, 27 × 20 mm cystic area in myometrium has been detected. Beta hcg <1 mIU/mL value was reached three months later, and this cystic area resolved spontaneously. Systemic methotrexate administration combined with ultrasound-guided local methotrexate injection into the gestational sac might be considered as the first-line treatment in the management of hemodynamically stable patients having cornual pregnancy even with high beta HCG values and risk of myometrial cystic formation.

## 1. Introduction

Cornual pregnancy is an uncommon form of ectopic pregnancy and represents 1%–6% of all ectopics [1, 2]. Diverse therapeutic options exist for the management of this pathology. Surgical management has been the treatment of choice which involves either laparoscopy or laparotomy and either cornual resection or hysterectomy. Medical treatment involves MTX or KCL administration and is associated with treatment failures that may result in uterine rupture and life-threatening hemorrhage. Therefore, when the medical treatment is considered, it is recommended in the case of a symptom-free patient with a beta HCG value <5000 IU/L with either systemic or ultrasound-guided injection of MTX to the cornual gestational sac [3]. However, ultrasound-guided puncture and injection carries the risk of bleeding or rupture at needle puncture site [4]. In this paper we want to report an interesting medical treatment complication of a cornual pregnancy case.

## 2. Case Report

**Figure 1:**
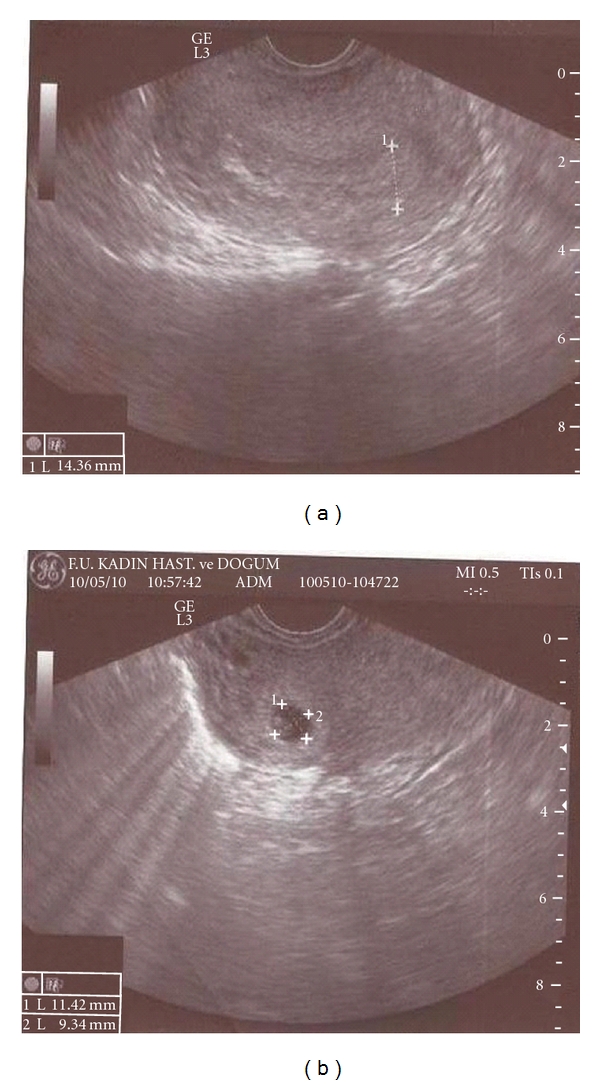
15 mm endometrial thickness and 11 × 9 mm cornual gestational sac.

**Figure 2:**
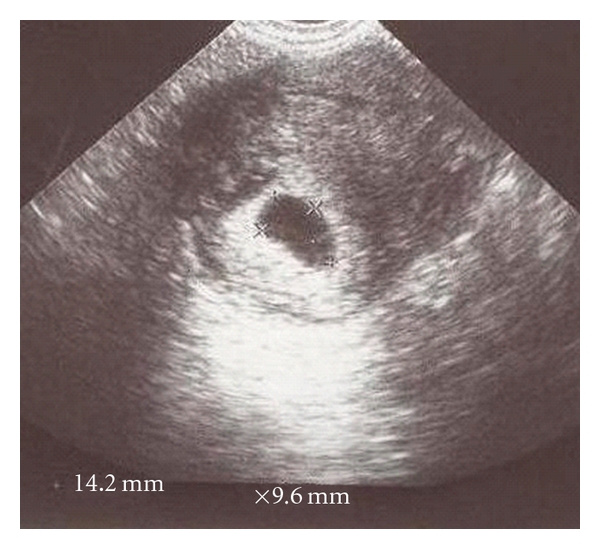
14 × 10 mm cornual gestational sac despite two systemic and one local MTX injection.

**Figure 3:**
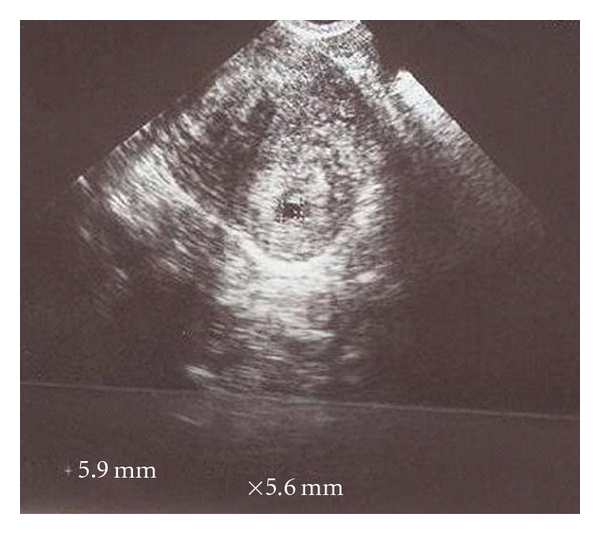
6 × 6 mm cornual gestational sac after second local and third systemic MTX injection.

**Figure 4:**
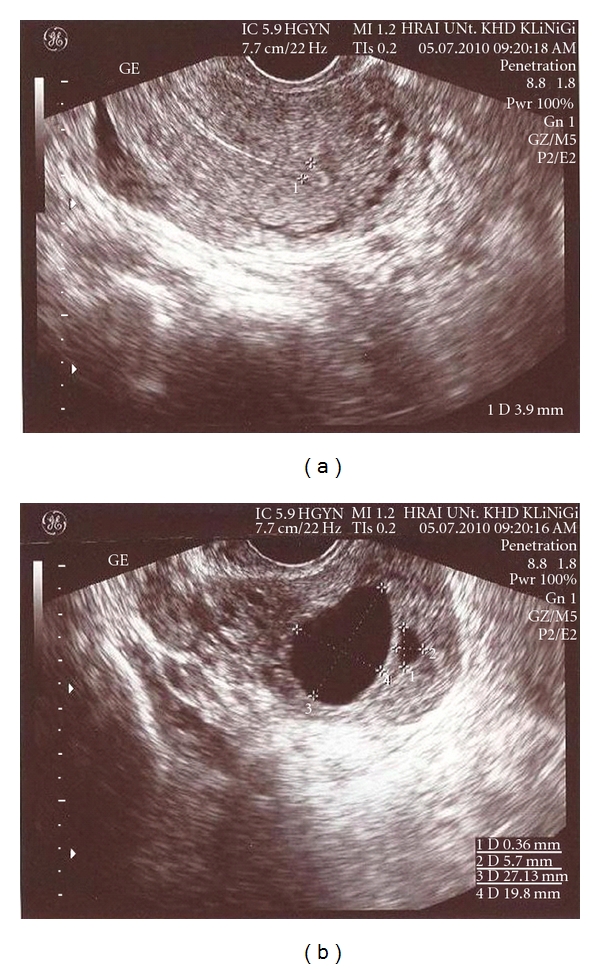
Regular endometrial ring and 27 × 20 mm myometrial cystic area near of first distorted 8 × 6 mm gestational sac residue.

A 39-year-old, gravida three, para two woman referred to our clinic because of suspected cornual pregnancy. She did not remember her last period date. The patient's systemic and gynecologic examination were normal, vital signs were within normal range, and she was hemodynamically stable. Our first beta HCG value was 22000 mIU/mL and TVUSG showed an empty uterine cavity, a 15 mm endometrial thickness, no free fluid in the cul-de-sac, and a right cornual gestational sac with a diameter of 11 × 9 mm including a suspicious embryonic pole with a yolc sac (Figure 1). Laboratory evaluation revealed normal complete blood count, liver and kidney functions, and no contraindication existed to MTX therapy. Hemoglobin level was 11 g/dL. After counseling the patient about the risks of medical treatment with MTX, an intramuscular systemic injection of 80 mg (50 mg/m^2^) MTX was administered. Four days after systemic MTX injection, beta HCG value increased to 27000 mIU/mL, patient was stable and had no complaint. Performance of a local MTX injection was decided. A 17-gauge double lumen follicle aspiration needle (Swemed, Vitrolife, Sweden) was inserted in the right lateral fornix and passed through the myometrium into the gestational sac under TVUSG guidance. Gestational sac content was aspirated and instead 10 mg MTX was injected into the sac under TVUSG guidance. The sac diameter after this procedure was observed to be 7 × 6 mm. Seven days after systemic MTX treatment, beta HCG value was still found to increase to 29984 mIU/mL. Second systemic 80 mg MTX injection was administered. Four days after second MTX injection, beta HCG value had a plateau at 29034 mIU/mL and cornual sac diameter was 7 × 6 mm. Seven days after second MTX injection, beta HCG value decreased to 22248 mIU/mL; however, gestational sac was observed to get enlarged to 14 × 10 mm dimensions (Figure 2). Second TVUSG-guided sac aspiration and 10 mg local MTX injection into the sac was performed together with the third systemic 80 mg MTX injection and folinic asid replacement. After the procedure the sac diameter was 6 × 6 mm (Figure 3). Four days after third systemic MTX injection, beta HCG value decreased to 18722 mIU/mL and seven days after third systemic MTX injection the value progressively decreased to 13029 mIU/mL. The patient was stable and she was discharged with weekly beta HCG measurements. On the second week of followup, two regular cystic areas within the myometrium of 18 × 11 mm and 9 × 8 mm in the right cornual region were observed although beta HCG value continued to decrease. On the third week of followup, the bigger cystic area reached 27 × 20 mm diameter; however, patient had no complaint and was stable (Figure 4). Because of decreasing beta HCG values, no surgical intervention was performed. After three months of followup, both cystic areas showed spontaneous resorption, and beta HCG value decreased to <1 mIU/mL. We have obtained the written permission of this presented case.

## 3. Discussion

Cornual pregnancy is an uncommon form of ectopic pregnancy and represents 1%–6% of all ectopics [1, 2]. Diverse therapeutic options exist for the management of this pathology. Surgical management has been the treatment of choice which involves either laparoscopy or laparotomy and either cornual resection or hysterectomy. Medical treatment involves MTX or KCL administration and is associated with treatment failures that may result in uterine rupture and life-threatening hemorrhage.

Methotrexate is a chemotherapeutic agent which acts as a trophoblast growth inhibitor by inhibiting DNA synthesis and can be given either as a local injection to the gestational sac or systemically, either as a single intramuscular dose or multiple doses [5]. Systemic administration of MTX is widely used in nearly all forms of ectopic pregnancy in patients with stable vital signs. However, TVUSG-guided local MTX injection to the cornual gestational sac has been presented as case reports and suggested to be a safe and effective alternative to surgical and systemic MTX therapy [2, 5]. Especially in live ectopic gestations, systemic MTX should not be the first-line treatment due to high failure rates (30%), surgery, or local MTX injections into the sac should be considered [5]. With this paper, we presented the successful combined use of systemic and TVUSG-guided local MTX injection in the management of a cornual pregnancy with a high initial beta HCG titer. 

In a previous report, a cornual pregnancy was successfully treated with a 100 mg local MTX injection after a failed response to three-dose systemic 100 mg MTX administration [6]. Local MTX injection was suggested as a good therapeutic option in the management of an unruptured cornual pregnancy after failed systemic MTX administration. Another author similarly suggested the use of local MTX administration of 1 mg/kg body weight under TVUSG guidance or with laparoscopy to be effective in the management of unruptured cornual pregnancies [7]. Another investigator presented a cornual pregnancy with a coexistent intrauterine pregnancy and successfully treated cornual sac with aspiration under ultrasound guidance followed by local injection of 12.5 mg MTX [8]. In the present case, we combined both modalities, local and systemic MTX administration, which is the first report of such an approach. In the literature, successful treatment of cornual pregnancy with just a single dose of MTX has been reported [9, 10]. Medical treatment with a single MTX injection has been recommended as an alternative to surgical treatment of cornual pregnancy. However, it is associated with significantly increased risk of failure, subsequent uterine rupture and emergency surgery [10]. In the present case, both because the initial beta HCG value was high and also serial beta HCG measurements did not show expected decline, multiple systemic and local MTX injections were administered. Cornual pregnancy with an initial beta HCG value of less than 5000 mIU/mL is usually treated successfully with single-dose MTX, but when the value is greater than 5000 mIU/mL, more than a single dose is usually required and failure or complication is more likely [1]. However, upper limit of beta HCG value at which medical treatment with MTX will fail is not clear. 

For local administration of MTX, various injected doses were given in the literature like 12.5 mg, 25 mg, and 100 mg. In our case, we aspirated the sac fluid and injected 10 mg MTX instead. Administration of a higher dose would have eliminated the need for multiple local injections which deserves further investigation. One disadvantage of minimally invasive or medical treatment modalities is a higher incidence of recurrence [11]. However, our case is a multiparous woman and just wanted to preserve her uterus; she did not plan to get pregnant in the future. 

With the present case, we further want to share our experience in the medical treatment of cornual pregnancy with a high beta HCG value. We initiated medical treatment with one cornual sac, but during followup, despite decreasing beta HCG values, a new cystic area was observed which might be a local reaction of TVUSG-guided injection. In the literature, increase in the size of the gestational sac after injection was reported in some cases; however, appearance of a new cystic area has not been described [5]. This was the first report of such a sonographic appearance following local MTX injection into the cornual gestational sac. Another explanation to this condition might be an unnoticed initial twin cornual pregnancy which is less likely. We performed the sonography with two dimensional TVUSG and use of imaging techniques like three-dimensional ultrasound and magnetic resonance imaging might help to delineate such cases more precisely. Of course, we could not confirm cystic myometrial formation as lack of histopathologic evaluation. 

In conclusion, combined use of systemic and TVUSG-guided local MTX in the management of cornual pregnancy is a safe and effective treatment method in hemodynamically stable patients even in the presence of an initial high beta HCG value. Therefore, it might be considered as an initial therapeutic option in unruptured cornual pregnancy before proceeding to laparotomy or laparoscopy with hysterectomy or cornual resection. And myometrial cystic formation should be kept in mind during local methotrexate applications.
